# HIV-1 infection induces changes in expression of cellular splicing factors that regulate alternative viral splicing and virus production in macrophages

**DOI:** 10.1186/1742-4690-5-18

**Published:** 2008-02-04

**Authors:** Dinushka Dowling, Somayeh Nasr-Esfahani, Chun H Tan, Kate O'Brien, Jane L Howard, David A Jans, Damian FJ Purcell, C Martin Stoltzfus, Secondo Sonza

**Affiliations:** 1 Macfarlane Burnet Institute for Medical Research and Public Health, Melbourne, Victoria, Australia; 2 Department of Microbiology and Immunology, University of Melbourne, Melbourne, Victoria, Australia; 3 Department of Biochemistry and Molecular Biology, Monash University, Melbourne, Victoria, Australia; 4 Department of Microbiology, University of Iowa, Iowa City, Iowa, USA; 5 Department of Microbiology, Monash University, Melbourne, Victoria, Australia

## Abstract

**Background:**

Macrophages are important targets and long-lived reservoirs of HIV-1, which are not cleared of infection by currently available treatments. In the primary monocyte-derived macrophage model of infection, replication is initially productive followed by a decline in virion output over ensuing weeks, coincident with a decrease in the levels of the essential viral transactivator protein Tat. We investigated two possible mechanisms in macrophages for regulation of viral replication, which appears to be primarily regulated at the level of *tat *mRNA: 1) differential mRNA stability, used by cells and some viruses for the rapid regulation of gene expression and 2) control of HIV-1 alternative splicing, which is essential for optimal viral replication.

**Results:**

Following termination of transcription at increasing times after infection in macrophages, we found that *tat *mRNA did indeed decay more rapidly than *rev *or *nef *mRNA, but with similar kinetics throughout infection. In addition, *tat *mRNA decayed at least as rapidly in peripheral blood lymphocytes. Expression of cellular splicing factors in uninfected and infected macrophage cultures from the same donor showed an inverse pattern over time between enhancing factors (members of the SR family of RNA binding proteins) and inhibitory factors (members of the hnRNP family). While levels of the SR protein SC35 were greatly up-regulated in the first week or two after infection, hnRNPs of the A/B and H groups were down-regulated. Around the peak of virus production in each culture, SC35 expression declined to levels in uninfected cells or lower, while the hnRNPs increased to control levels or above. We also found evidence for increased cytoplasmic expression of SC35 following long-term infection.

**Conclusion:**

While no evidence of differential regulation of *tat *mRNA decay was found in macrophages following HIV-1 infection, changes in the balance of cellular splicing factors which regulate alternative viral pre-mRNA splicing were observed. These changes correlated with changes in Tat expression and virus production and could play an important role in viral persistence in macrophages. This mechanism could provide a novel target for control of infection in this critical cell type, which would be necessary for eventual eradication of the virus from infected individuals.

## Background

Macrophages are one of the major target cells for HIV-1 in the body, are infected very early and remain an important reservoir of long-lived cells [1]. Even prolonged, highly active antiretroviral therapy (HAART) is unable to clear infection from cells of the macrophage lineage, as we [2] and others [3,4] have shown, due to a combination of the reduced efficiency of most antiretroviral drugs in these cells and their location in poorly accessible tissue sites such as the brain [5]. The proportion of tissue macrophages harbouring HIV-1 may be as high as 50% [6] and they become a major source of virus during opportunistic infection [1] or when CD4+ T cells are depleted [7]. Also, virus found in plasma of patients on HAART is more likely to be closely related to that in monocytes than to either activated or resting CD4+ T cells [4]. In the gut-associated lymphoid tissue, the largest lymphoid organ in the body and the primary site for acute HIV-1 replication [8], macrophages are the predominant viral reservoir following massive depletion of CD4+ memory T cells during this acute infection stage [9]. Additionally, infection impairs vital macrophage functions such as phagocytosis, intracellular killing, cytokine production and chemotaxis [10].

Unlike infection in activated primary CD4+ T cells or cell lines, HIV-1 infection of macrophages is not generally lytic. Due to the inherent difficulties of accessing tissue macrophage sources or patient specimens, limited work has been done in characterising HIV-1 infection in macrophages *in vivo*. The monocyte-derived macrophage (MDM) model has therefore been used extensively for this purpose [11-13]. In this system, monocytes isolated from peripheral blood are differentiated in culture and then infected with macrophage-tropic (mostly CCR5 coreceptor-using or R5) isolates and strains of HIV-1. These infected cells can be monitored for long periods (months) without significant depletion due to cell lysis and remain infected for the duration of culture. Productive infection in this system increases relatively slowly for 2–3 weeks compared to that in primary activated PBMC cultures (cell donor and viral strain dependent), before beginning to decline progressively over the ensuing few weeks [13]. Preceding the decline in virus production by several days, we have found a specific progressive decline in the expression of mRNA encoding the essential viral regulatory protein, Tat, the viral transactivator which controls transcription. Providing Tat exogenously restores virus production [13].

While significant attention has been paid in recent times to the resting memory CD4+ T cell reservoir of HIV-1, much less effort has been directed to tackling other cellular reservoirs such as cells of the macrophage lineage (monocytes, macrophages, microglia, dendritic cells etc.). Without also clearing HIV-1 infection from these long-lived cells, eradication of the virus from infected individuals is not achievable, since these cells will simply reseed other susceptible cells. For this reason, we investigated two possible mechanisms responsible for the decrease in *tat *mRNA and subsequent decline in virus production in MDM.

Firstly, the stability of the *tat *mRNAs or the pathways leading to their degradation may change with time in these cells or be altered by infection with HIV-1. Differential mRNA stability, in which the levels of transcripts are regulated by controlling the rate at which they decay, is a common cellular mechanism for regulating expression of particular genes, such as those for cytokines and growth factors, and provides the cell with flexibility in effecting rapid change (reviewed recently by Garneau and colleagues [14]). Additionally, some viruses, especially herpesviruses, can regulate the degradation of both host and viral mRNAs to help redirect the cell from host to viral protein synthesis and facilitate sequential expression of viral genes [15].

An alternative explanation for the pattern of *tat *mRNA expression during long-term infection in MDM involves regulation of alternative splicing. In contrast to the alternative splicing of most cellular mRNAs, processing of the HIV-1 primary transcript or pre-mRNA results also in cytoplasmic accumulation of incompletely and unspliced viral mRNAs that are necessary for the expression of Env, Vif, Vpr, Vpu and the *gag *and *pol *gene products respectively. Unspliced mRNA serves also as genomic RNA that is encapsidated within progeny virions. Completely spliced viral mRNAs, which are detected earliest following infection, are required for expression of the regulatory viral proteins Tat, Rev and Nef. More than 40 unique incompletely and completely spliced mRNAs are generated through alternative splicing of the primary transcript [16] and changes to this highly regulated system can have dramatic effects on the efficiency of replication.

**Figure 1:**
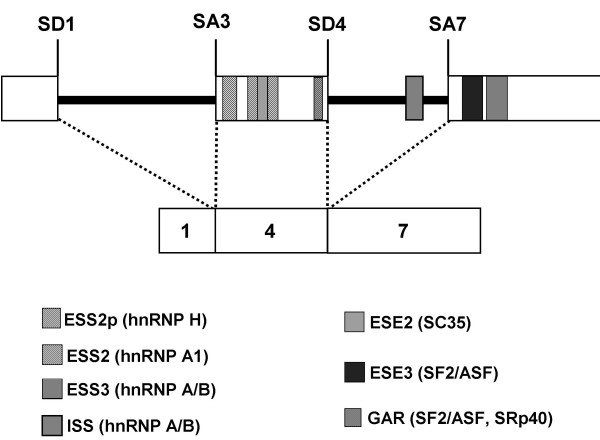
**Splicing regulation of the tat gene**. Schematic of Tat pre-mRNA and the Tat1 spliced product (containing exons 1, 4 and 7) as an example, showing positions of introns (thick black line) and exons (large open boxes), splice donors (SD) and acceptors (SA), exonic splicing enhancers (ESE, dotted boxes), exonic and intronic splice silencers (ESS, hatched boxes; ISS, grey box) and the GAR splice enhancer (checkerboard box). Splicing factors which bind these splicing regulatory elements are given for each in parentheses. NB: ESE2 element is contained within ESS2 and this region contains overlapping binding sites for SC35 and hnRNP A1. Not to scale.

HIV-1 splicing is regulated in part by cellular splicing factors that function via both positive and negative *cis *elements within the viral genome that act to promote or repress splicing. To date, 4 exonic splicing silencers (ESS) and 1 intronic splicing silencer (ISS) have been identified within the viral genome, together with 3 exonic splicing enhancers (ESE) [reviewed in 17] and a GAR splicing enhancer [18]. In general, members of the serine-arginine rich (SR) family of phosphoproteins bind to enhancer elements and promote use of nearby splice sites, while members of the heterogeneous nuclear ribonucleoprotein (hnRNP) family of splicing factors bind silencer elements and inhibit splice site utilisation [17]. The enhancer and silencer elements and relevant splice donor (SD) and splice acceptor (SA) sites involved in *tat *mRNA splicing, together with the relevant cellular factors involved, are shown in Figure 1. The SR proteins ASF/SF2, SC35, SRp40 and 9G8 have been implicated as positive splicing factors that affect Tat expression, while hnRNPs H and members of the A/B family are thought to inhibit splicing of *tat *mRNA [18-25].

To better understand the mechanism underlying the regulation of HIV-1 replication in macrophages, we determined the relative stability of *tat *mRNA in MDM at increasing times after infection and the effect of infection on the expression of cellular splicing factors. While we found no evidence for differential mRNA stability of *tat *mRNA in macrophages at any time after infection, changes in the balance between enhancing and inhibitory cellular splicing factors induced by infection suggest that regulation of HIV-1 alternative splicing plays a key role in persistent infection in these important viral reservoirs. This might provide a novel target for control of HIV-1 in macrophages.

## Results

### Stability of tat mRNA in MDM compared to PBL

Our preliminary findings on differential *tat *mRNA expression during the course of long-term infection in MDM suggested the possibility that the stability of the *tat *message itself or the pathways leading to its degradation may change with time in these cells or be altered by infection with HIV-1. We therefore determined the stability of *tat *mRNA relative to transcripts encoding the two other main HIV-1 regulatory proteins, Rev and Nef, which are expressed at high levels throughout infection in MDM [13].

**Figure 2:**
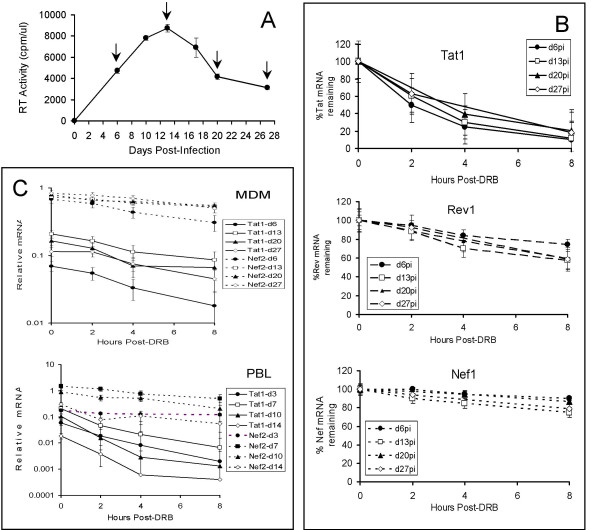
**Stability of HIV-1 regulatory gene mRNAs in primary macrophages and T cells**. **A**: Replication kinetics of HIV-1_Ba-L _in MDM from a representative donor. Arrows show times at which DRB was added to terminate transcription. **B**: Relative decay curves for Tat1, Rev1 and Nef1 mRNAs in MDM following addition of DRB as determined by RT-PCR, PAGE and densitometry. Results are expressed as a percentage of levels present immediately after addition of DRB. Mean + SEM from 6 donors. **C**: Stability of Tat1 and Nef2 mRNA compared to GAPDH mRNA following DRB addition at increasing times after infection in MDM and PBL from the same donor, as determined by real-time PCR. Mean + SEM from 3 donors.

Following infection with the macrophage-tropic, R5 strain Ba-L, MDM from individual donors were treated at approximately weekly intervals with the RNA pol II inhibitor DRB to terminate transcription in the cells. A typical growth curve for Ba-L in MDM, with times when transcription was terminated indicated, is shown in Fig. 2A. The decay of specific representative mRNA transcripts was then measured by RT-PCR, with products being detected by ^32^P-labelling, separation on sequencing gels and densitometry (Fig. 2B). Tat1 and Rev1 mRNAs were chosen for analysis because they are the most abundant of the *tat *and *rev *messages in infected MDM, in which only minimal expression of other exonic forms are detectable [13]. Nef1 mRNA was analysed because it is expressed at similar levels to Tat1, whereas Nef2 is expressed at such high levels in MDM [13] that it was difficult to reliably quantify by densitometry.

We found that Tat1 mRNA did indeed decay more rapidly in MDM than Rev1 and much more rapidly than Nef1 mRNA, by ~80% within 8 hrs compared to ~30% and ~10–20% respectively. However, the rate of decay was similar for all mRNAs throughout the 4 weeks over which the cultures were followed (Fig. 2B). Tat1 mRNA was found to be, if anything, less stable in PBL than in MDM from the same donor. Again, however, it decayed at a similar rate, as measured this time by real-time RT-PCR, regardless of time after infection (Fig. 2C). As was found in MDM, *nef *mRNA was also much more stable in PBL than *tat *mRNA. For real-time RT-PCR analysis, Nef2 message was used due to the difficulty of designing primers specific for Nef1 which were functional in this technique. By the above RT-PCR/gel technique however, Nef1 and Nef2 were consistently found to be of similar stability. *Tat *mRNA expression levels in MDM during long-term infection *in vitro *did not, therefore, appear to correlate with differential decay rates since *tat *mRNA was not less stable later in infection than earlier as would be predicted from the replication kinetics in MDM (Fig. 2A).

### Effect of HIV-1 infection on expression of cellular splicing factors in macrophages

Since differential mRNA stability did not appear to explain the Tat-dependent nature of regulation of HIV-1 replication in MDM, we investigated the effects of infection on the cellular splicing machinery, specifically the expression of particular enhancing and inhibitory splicing factors implicated in viral alternative splicing.

### hnRNP expression

**Figure 3:**
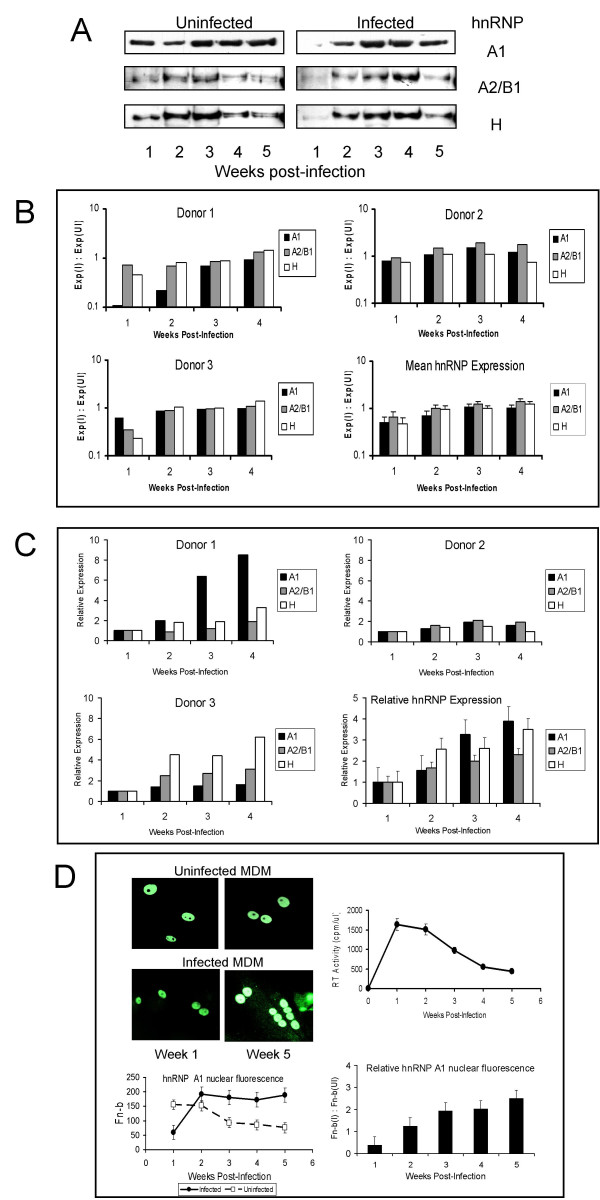
**Expression of hnRNPs in MDM**. **A**: Western blot detection of hnRNPs A1, A2/B1 and H in nuclear extracts from uninfected and HIV-infected MDM from the same donor (donor 1) over a 5-week period following infection. Representative of 6 cultures. **B**: Quantification by densitometry (Odyssey Infra-red Imaging System, Li-Cor) of hnRNP expression in 3 representative MDM cultures (Donors 1–3) following infection relative to expression in matched uninfected cells at each time point (Exp [I] : Exp [UI]), showing the donor-to-donor variability commonly seem with MDM cultures. Mean expression levels are shown in the bottom right hand panel (mean + SEM of 6 donor cultures). **C**: Data from B above normalized to expression levels at 1 week post-infection. D: Confocal laser scanning microscopy of hnRNP A1 expression in the nuclei of MDM, from a different donor from those used in A and B above, exposed to virus or mock infected, 1 and 5 weeks after infection. The replication kinetics for this donor are shown in the top right hand panel. Specific nuclear fluorescence (Fn-b) of at least 10 cells per chamber at weekly intervals after infection was determined using Image J software after subtracting the background fluorescence from cells stained without primary antibody (mean + SEM; bottom left hand panel). The nuclear fluorescence of infected cells (Fn-b [I]) relative to uninfected cells (Fn-b [UI]) over the 5 weeks of infection for this culture is also shown (mean + SEM; bottom right hand panel). Representative of 6 donors.

Although quite donor variable in abundance, nuclear expression of hnRNPs was generally found to vary over time in culture in MDM, with peak levels usually reached 3–4 weeks after isolation of the cells, followed by a gradual reduction with further culture. HIV-1 infection was found to initially decrease the expression of hnRNPs, with the magnitude of this effect variable between donors even though both the virus inoculum and time of infection of the cells was kept constant for all cultures (Fig. 3A). Levels of hnRNP A1, A2/B1 and H all remained depressed for 1 to 2 weeks following infection, compared to uninfected cells from the same donors treated similarly, before returning to levels comparable to or slightly higher than those in corresponding control cells around the peak of infection (2–4 weeks; Fig. 3B). Compared to the first week following infection, relative hnRNP expression in infected MDM increased by 2–4 fold over the next month of culture (Fig. 3C).

When a separate group of MDM cultures were examined by confocal laser scanning microscopy (CLSM; Fig. 3D), expression in infected cultures was clearly reduced at early time points compared to that in the uninfected control cultures from the same donor (~3-fold reduction in specific mean nuclear fluorescence [Fn-b] of hnRNP A1 one week after infection in donor shown; Fig. 3D – bottom left hand panel). From the peak of infection (1 week post-infection in donor shown in Fig. 3D), hnRNP expression begins to increase to levels above those seen in uninfected cells from the same donor maintained in culture for the same period. After a further month of culture, corresponding to 5 weeks post-infection, hnRNP expression in control cells had diminished somewhat but in infected cells it was generally increased over that seen earlier in infection in the same cells, as well as considerably higher than that seen in the uninfected cells at the same time (relative nuclear fluorescence in infected cells (Fn-b [I]) approximately 2.5-fold higher than in uninfected cells (Fn-b [UI]); Fig. 3D, bottom right hand panel). Throughout infection in macrophages, hnRNP expression remained highly localised in the nucleus, with no consistent evidence of increased shuttling to the cytoplasm.

### SR protein expression

**Figure 4:**
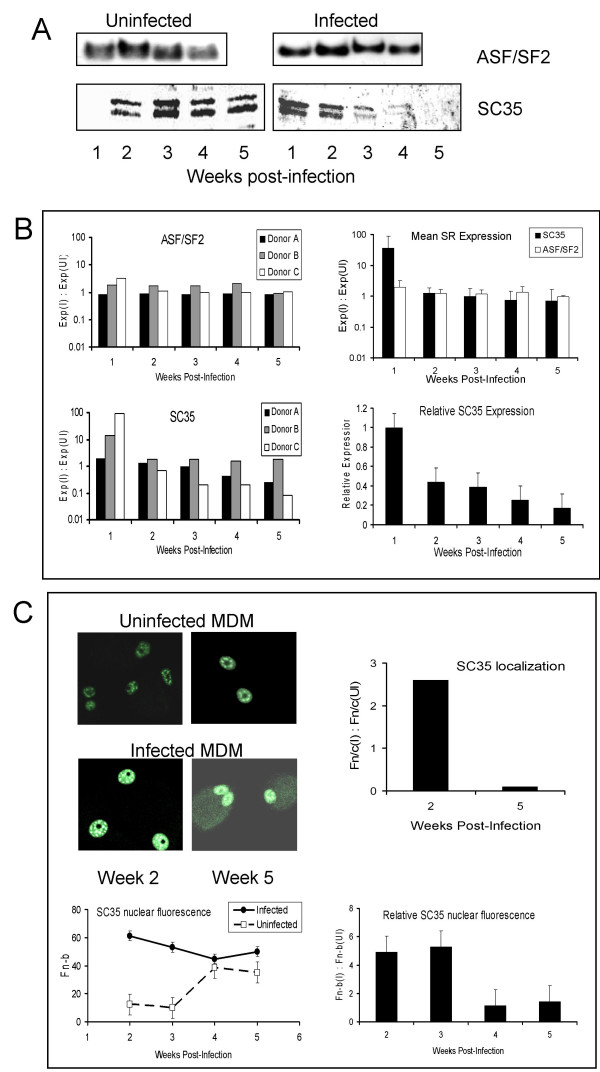
**Expression of SR proteins in MDM**. **A**: Western blot detection of ASF/SF2 and SC35 in nuclear extracts from uninfected and HIV-infected MDM over a 4–5 week period following infection. Representative of 6 cultures. **B**: Densitometric analysis as in Fig. 3 of Western blot expression of SC35 and ASF/SF2 in 3 representative infected MDM cultures (Donors A-C; black, grey and white bars respectively) relative to uninfected cells (Exp [I] : Exp [UI]) at each time point. Mean expression levels are shown in the top right hand panel (mean + SEM of 6 cultures). Relative SC35 expression, normalized to levels at 1 week post-infection, is shown in the bottom right hand panel. **C**: Confocal scanning laser microscopy of SC35 expression in the nuclei of uninfected and infected MDM 2 and 5 weeks after infection of a separate donor culture from those used in A and B above. Specific nuclear fluorescence (Fn-b) for infected and uninfected cells (bottom left hand panel) and relative nuclear fluorescence (Fn-b [I] : Fn-b [UI], bottom right hand panel) over that period, as per Fig. 3, are shown. Top right hand panel shows the ratio of nuclear to cytoplasmic fluorescence (Fn/c) of infected cells relative to uninfected cells (Fn/c [I] : Fn/c [UI]) for weeks 2 and 5. Fn/c > 1 indicates predominantly nuclear localisation, while Fn/c < 1 represents predominantly cellular localisation. Representative of 6 donors.

Expression of SR proteins was also found to vary over time in culture in MDM, with again considerable donor variability. While ASF/SF2 was expressed at similar levels throughout the time course, SC35 was initially low, increased over 2–3 weeks then declined again (Fig. 4A – left hand panels). HIV-1 infection had little, if any, effect on ASF/SF2 expression but markedly increased SC35 expression in the nucleus in the first week and sometimes longer following infection (Figs. 4A – right hand panels, and 4B – left hand panels). After the first week in infected cultures, SC35 expression declined progressively for the remainder of the time course, both when compared to levels in uninfected cells at the same time point (Fig. 4B – top right hand panel) and even more so when compared to levels in infected cells at week 1 (Fig. 4B – bottom right hand panel). This pattern was found irrespective of when the peak of replication was reached in each particular donor (from 1 to 3 weeks after infection in the donors analysed). By CLSM (Fig. 4C – top left hand panel), SC35 was more strongly expressed in HIV-infected MDM cultures, in characteristic nuclear speckles, than in the matched uninfected cells in the first few weeks following infection (~5-fold increase in relative nuclear fluorescence, i.e. mean nuclear fluorescence of infected relative to uninfected cells [Fn-b(I) : Fn-b(UI)], at 2 and 3 weeks pi in donor shown; Fig 4C – bottom right hand panel). By 4–5 weeks, SC35 expression in uninfected cells had increased, although not to the levels seem in infected cultures in the first week or two of infection. In infected cultures it had declined by 5 weeks compared to early time points, to levels similar to those in uninfected cells (Fig. 4C – bottom left hand panel). As expected, SC35 was expressed almost exclusively in the nucleus of uninfected cells throughout the time course and in infected cultures early in infection, with a relative nuclear : cytoplasmic ratio (Fn/c [I] : Fn/c [UI]) of ~2.5 at 2 weeks p.i. indicating strong nuclear localisation (Fig. 4C – top right hand panel). Interestingly, cytoplasmic expression appeared to increase with time after infection with HIV-1 in MDM, as shown by the substantial decrease in the relative nuclear : cytoplasmic ratio to well below 1 by 5 weeks p.i. (Fig. 4C – top right hand panel). ASF/SF2 expression in macrophages changed little either during culture or following infection (not shown).

### mRNA expression

**Figure 5:**
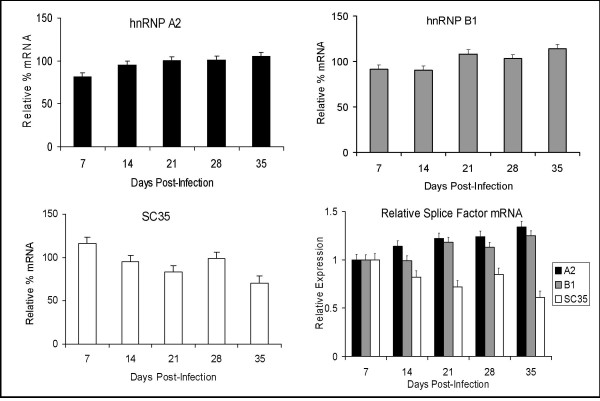
**Effect of HIV-1 infection on splicing factor mRNAexpression in MDM**. mRNA was extracted from uninfected and HIV-infected MDM from individual donors (distinct from those used in Figs. 3 and 4) at weekly intervals over a 5 week period. RT-PCR for splicing factor message levels was performed following standardisation for GAPDH mRNA. Expression of splicing factor mRNA in infected MDM is given as a percentage of that in matched uninfected cells at each time point. Results for the representative hnRNPs A2 and B1 and the SR protein SC35 are shown (mean + SEM of 3 donors). Also shown is the relative expression normalised to mRNA levels at 7 days post-infection (bottom right hand panel).

To determine whether the changes in splicing factor expression in MDM following infection with HIV-1 were reflected at the message level, mRNA was extracted from uninfected and infected MDM from individual donors over a 5-week period and RT-PCR performed for splicing factors. While again there was considerable variation in mRNA expression between donors and over time in culture, hnRNP mRNA levels were generally reduced by 10–20% in the first week following infection, before recovering to levels similar to those in uninfected controls or slightly higher by 3–4 weeks after infection (Fig. 5 – top panels). SC35 mRNA was expressed at higher levels in infected cells a week after infection (~10% greater than matched control cells), then declined to below that found in the control cells over the next 2–3 weeks of infection (Fig. 5 – bottom left hand panel). The inverse pattern of expression after the first week of infection for hnRNPs, which increase, compared to that for SC35, which decreases, was also evident at the level of mRNA (Fig. 5 – bottom right hand panel).

## Discussion

Differential mRNA stability is known to be a common mechanism by which cells can rapidly alter expression rates of particular proteins such as cytokines, growth factors and proto-oncogenes [14], and it has also been shown to be used by several viruses to regulate expression of both viral and cellular genes [15,26]. However, we found no evidence that this mechanism is involved in the regulation of the essential HIV-1 regulatory protein Tat in macrophages. From our previous work, Tat appears to play a central role in the replication of HIV-1 in this cell type. Tat expression increases initially in macrophages, leading to increased virus production, but then declines over several weeks, heralding a reduction in productive infection [13]. This reduction in Tat is not seen during the decline in productive infection in primary T cells from the same donor, which is due to cell lysis and exhaustion of the pool of susceptible cells in the culture. Unlike T cells, macrophages are relatively refractory to the cytopathic effects of HIV-1 and remain infected for their life span. Although we did indeed find that *tat *mRNA decayed more rapidly in macrophages than other regulatory messages, it also decayed at a similar or faster rate in primary T cells. Additionally, this accelerated decay rate remained relatively constant throughout the infection period followed in both cell types (5–6 weeks in macrophages and 3 weeks in T cells).

Since we and others [17-25,27-34] have shown that control of HIV-1 gene expression is heavily regulated at the level of mRNA splicing through the binding of numerous cellular splicing factors at multiple enhancing and silencing elements, and the *tat *gene is particularly rich in these elements, we reasoned that this may be of particular importance in macrophages. Therefore, we investigated the changes in splicing factor expression induced by infection in macrophages. With no previous reports on the expression of cellular splicing factors of the hnRNP or SR families in macrophages, we determined how these varied during long-term culture and what effect viral replication had on them. While we found considerable, but not unexpected, donor-to-donor variability in expression of hnRNPs A1, A2/B1 and H, the major splicing inhibitory factors implicated in binding HIV-1 pre-mRNA [20-23,25], and in SC35 and ASF/SF2, SR proteins similarly implicated in enhancing splicing [24,25,27,28], some general patterns emerged.

While hnRNP proteins were expressed at relatively similar levels in the nucleus of MDM over the course of 6 weeks in culture, HIV-1 infection resulted in a transient reduction in expression in the first week before the levels recovered to those in uninfected cells or slightly higher over the next week or two. In contrast, SC35 nuclear expression, quite low in macrophages in the first week or two of culture, was dramatically up-regulated early following infection before returning to uninfected cell levels or lower later. Although the changes seen at the mRNA level were not pronounced, they followed the same general pattern as seen at the protein level, namely higher expression of SC35 and lower expression of hnRNPs early, followed by recovery of hnRNP expression and declining SC35 expression. HIV-1 infection appeared to have a specific effect on SC35 expression rather than a general effect on all SR proteins since ASF/SF2 expression remained at similar levels throughout, regardless of infection.

Although no other studies on the effects of HIV-1 replication on splicing factors in primary macrophages or even monocyte/macrophage cell lines have been reported, there is some evidence of changes in expression levels in T cells during infection. A 2- to 3-fold increase in expression of SC35 was observed by the second day of infection of H9 cells [35], while conversely, 9G8 mRNA was down-regulated after 60 hours infection of MT-4 cells [36]. The effects of changes in these factors on HIV-1 alternative splicing has primarily been elucidated by over-expression studies. Over-expression of SC35 or SRp40 in HeLa cells co-transfected with a *gag/pol*-deleted HIV-1 plasmid resulted in specific increases in *tat *mRNA of 3- to 4-fold [24], while over expression of ASF/SF2, SC35 and 9G8 in 293T cells transfected with pNL4-3 caused a large reduction in genomic RNA and structural proteins, with resultant substantial decreases in virion production [27]. In this latter study, each SR protein modified the viral RNA splicing pattern in a specific way such that ASF/SF2 increased the level of *vpr *mRNA, while SC35 and 9G8 caused a large increase in *tat *mRNA. The SC35 over expression study would support our findings of increased SC35 expression during the first week or two of macrophage infection having an impact on Tat expression. Expression and replication of other viruses, including human papillomavirus type 16 and adenovirus, have also been shown to be influenced by SR proteins [37,38].

Taken together, the results found for cellular splicing factors in macrophages suggest that HIV-1 infection changes the splicing factor milieu in these cells in such a way that might be expected to aid virus replication in the first week or two i.e. increased levels of splicing enhancing factor(s), including SC35 but probably not ASF/SF2, and lower levels of the competing inhibitory hnRNPs A/B and H, leading to increased *tat *mRNA expression and ultimately virus production. After two to four weeks of infection, depending on the individual donor, the environment in the nucleus of macrophages appears to be less favourable to the splicing of *tat *mRNA, with restored levels of hnRNPs and reduced levels of SC35, coinciding with reduced virus output. This scenario agrees with the results of Jacquenet and colleagues, which strongly suggest that changing the hnRNP/SC35 balance in cells leads to activation or repression of splicing at site SA3, thus regulating Tat expression [27].

The reduction in nuclear expression of SC35 seen after several weeks of infection in MDM may be, in part, due to its translocation to the cytoplasm. Whereas at 2 weeks post-infection SC35 was expressed almost exclusively in the nucleus of both infected and uninfected MDM, by 5 weeks higher levels where found in the cytoplasm of infected cells, as seen by the marked decrease in the nuclear : cytoplasmic ratio (Fn/c, Fig 4C). SC35 is not usually thought to traffic between the two cellular compartments, unlike some other SR proteins such as ASF/SF2 and hnRNP splicing factors including A1, because of its dominant nuclear retention signal in the RS domain [39]. HIV-1 also increases SC35 mRNA and protein expression following infection of the H9 T cell line, however in these cells, its cellular distribution does not appear to be altered [35]. In macrophages, infection may affect the nuclear import/export pathways used by SC35, such as the transportin-SR system [40] or, alternatively, the reversible phosphorylation of the RS domain that regulates the subcellular localisation and shuttling of SR proteins [41]. In contrast, HIV-1 infection did not appear to affect hnRNP A1 shuttling in our macrophage model, with no consistent evidence of increased cytoplasmic localisation in the first week of infection when nuclear expression was clearly reduced dramatically, nor later in infection when A1 levels had recovered. Cell stress has been shown to increase hnRNP A1 phosphorylation, resulting in its accumulation in the cytoplasm [42], but HIV infection does not appear to have a similar effect, at least not in MDM. If HIV-1 induces translocation of SC35 to the cytoplasm following long term infection, by whatever mechanism, less SC35 is then available to enhance splicing and, together with elevated levels of hnRNPs, the balance between splicing enhancement and inhibition would be altered to favour decreased *tat *mRNA expression and subsequent virus production.

Regulation of alternative splicing by interaction with the cellular splicing machinery may therefore be a mechanism by which HIV-1 is able to persist in macrophages, important and long-lived reservoirs of infection. This may also offer a novel target for therapies aimed at altering the expression of particular cellular splicing factors to control replication and spread from macrophages. One recently discovered class of splicing inhibitors, indole derivatives, have been shown to have a selective action on SR proteins, preventing their phosphorylation, which is required for activity [43]. Several indole derivatives were found to be potent inhibitors of HIV-1 RNA production in chronically infected, promonocytic U1 cells, by preventing or interfering with optimal alternative splicing [44]. The development of such agents which could selectively inhibit SR proteins, thus disturbing the hnRNP/SR balance that appears critical in the regulation of Tat expression and virus production in macrophages, might help control viral rebound from these reservoirs when therapy is interrupted or terminated and increased replication when CD4+ T cells have declined or during opportunistic infections. Used in conjunction with approaches aimed at clearing the T cell reservoir, they may offer hope for eventual eradication of HIV-1 from infected individuals.

## Conclusion

HIV-1 replication in macrophages appears to be regulated at the level of *tat *mRNA expression. Although *tat *mRNA is less stable than other regulatory gene messages, differential Tat expression during long-term infection in these cells is not due to changes in the rate of decay of its message, which appears to be relatively constant and similar to that in infected lymphocytes. However, changes in expression and possibly localisation of cellular splicing factors known to modulate viral alternative splicing do correlate with Tat levels and virus production in macrophages. HIV-1 infection initially up-regulates SC35 expression while down-regulating hnRNPs, thus altering the splicing factor balance within the cell to favour Tat expression. Around the time of peak virus production (about 2 weeks after infection), this change in splice factor balance is reversed and now favours inhibition of *tat *splicing, leading to reduced Tat expression and declining virus production, eventually to very low or undetectable levels. This 'latent' state may aid the persistence of HIV-1 in the macrophage reservoir. Manipulation of the splice factor balance to selectively induce or maintain macrophages in this non-productive state may represent a novel target for controlling macrophage infection and assisting in the eventual eradication of the virus from infected individuals, which cannot be achieved with current therapies.

## Methods

### Cell culture and HIV-1 infection

PBMC were isolated from buffy packs of HIV-seronegative donors provided by the Australian Red Cross Blood Service, Melbourne, by Ficoll-Hypaque (Amersham-Pharmacia) density gradient centrifugation. The cells were then further separated into a monocyte-enriched fraction and a monocyte-depleted, or PBL, fraction by plastic adherence as previously described [13]. Monocytes were cultured for 5 to 7 days to allow differentiation into macrophages in 6-well plates or 10 cm Petri dishes (Nunc) before being washed thoroughly to remove any remaining lymphocytes and infected with the R5 strain HIV-1_Ba-L _(1 RT unit/cell). Monocyte-derived macrophages from the same donors were mock infected and maintained similarly to the infected cells for use as controls. PBL were stimulated with PHA (2.5 ug/ml) for 2–3 days before infection with Ba-L (0.1 RT unit/cell) and culture in 25 cm^2 ^flasks in medium containing IL-2 (10 U/ml; Roche) as previously described [13]. Virus production was monitored by virion-associated reverse transcriptase activity in culture supernatants using the micro-RT assay as previously described [13].

### Termination of transcription and mRNA isolation

At weekly intervals for 4 weeks following infection of MDM, cells in 6-well plates were treated with DRB (5,6-dichloro-1-beta-d-ribofuranosylbenzimidazole; 20 uM final concentration in cell-culture medium) to terminate transcription from the HIV-LTR [45]. At 2, 4 and 8 hrs after addition of DRB, MDM were lysed and mRNA extracted using oligo(dT) magnetic beads according to the manufacturer's protocol (GenoVision). PBL cultured in 25 cm^2 ^flasks were similarly treated with DRB at twice-weekly intervals for 2 weeks and mRNA extracted. Isolated mRNA attached to the beads was then converted to cDNA using Superscript reverse transcriptase III (Invitrogen) according to the manufacturer's protocol and stored in 10 mM Tris-HCl, pH 7.5, until analysed by PCR (see below).

### PCR for HIV-1 spliced mRNA

cDNAs from infected and uninfected MDM and PBL were first standardized by real-time PCR using primers specific for GAPDH mRNA as previously described [46]. HIV-1 RNA expression profiles were generated from equivalent amounts of cDNA from infected MDM and PBL using primers Odp045 (487–498 of HXB2 genome, exon 1 and Odp032 (8507–8487, exon 7) [16], then relative expression of *tat*, *rev *and *nef *mRNAs determined by electrophoresis of radiolabelled products and phosphorimaging as described previously [13]. For some experiments, real-time PCR was used to quantify the relative expression of Tat1 and Nef2 mRNAs. cDNA was amplified using primers Odp423 (5'CGGCGACTGAATTGGGTG;735-743+5778-5786 [SD1-SA3])/Odp003 (5'GTCTCTCTCTCCACCTTCTTCTTC; 8447-8424) and Odp424 (5'CGGCGACTGGAAGAAGCG; 735-743+5977-5985 [SD1-SA5])/Odp003 respectively in a Sybr green reaction mix (BioRad). The amount of Tat1 and Nef2 in each sample was related to that of GAPDH mRNA.

### Immunoblotting

At weekly intervals from 1 week after infection, 2–5 × 10^6 ^infected and uninfected MDM from the same donors were lysed, nuclear extracts prepared and protein content determined as previously described [47]. Proteins from 10 ug extracts were then resolved by SDS-polyacylamide (12.5%) gel electrphoresis and transferred to nitrocellulose membrane (Hybond-C extra, Amersham-Pharmacia). Following blocking with 5% non-fat milk in PBS/0.1% Tween 20 (PBS-T), the membranes were probed with antibodies against hnRNPs A1, A2/B1 and H and SR proteins SC35 and ASF/SF2 (mouse monoclonal and goat polyclonal, all from Santa-Cruz Biotechnology) diluted in PBS-T containing 0.5% milk overnight at 4°C. Membranes were then extensively washed with PBS-T and incubated with biotin-conjugated secondary antibody (swine anti-goat, mouse, rabbit Ig, Dako) for 1 h at room temperature, washed again, then incubated with streptavidin-conjugated Alexa Fluor 680 (Invitrogen). Bands were analysed using the Odyssey Infra-red Imaging System (Li-Cor).

### Confocal scanning laser microscopy

Monocytes isolated as above were grown in 8-chambered plastic slides (Nunc), then half the chambers were infected with Ba-L after 5–7 days. At weekly intervals after infection, the cells were fixed with 4% paraformaldehyde for 1 h at 4°C, then blocked for 1 h at room temperature with 5 mg/ml BSA in PBS (PBS-B). They were incubated with anti-splicing factor antibodies (1:50 dilution in PBS containing 0.4% BSA and 0.4% Triton X-100 [PBS-BTX]) for 2 h at room temperature. The cells were then washed 5 times with PBS-B and incubated with secondary antibody conjugated to Alexa Fluor 488 (Molecular Probes), diluted 1:1000 in PBS-BTX, for 2 h at room temperature. Finally the cells were washed again as above and mounted with glycerol/propylgallate and a cover slip. Cells were viewed using a MRC 500 confocal laser scanning microscope (Bio-Rad) and analysed using Image J software (NIH).

### Splicing factor mRNA expression

mRNA from infected and uninfected MDM from the same donors was isolated, converted to cDNA and standardised as described above, then expression of splicing factor mRNAs determined by semi-quantitative PCR. Primers specific for SC35 (5'CTACAGCCGCTCGAAGTCTC-sense; 5'TTGGATTCCCTCTTGGACAC-antisense), hnRNP A2 (5'TCTCTCTCATCTCGCTCGGC-sense; 5'CTTACGGAACTGTTCCTTTTCTCTCT-antisense) and hnRNP B1 (5'TCTCTCTCATCTCGCTCGGC-sense; 5'CGGAACTGTTCCTTTTCTCTCTTT-antisense) were used in PCR reactions with HotStar Taq polymerase (Qiagen) for 35 cycles, the products separated on 2% agarose gels containing ethidium bromide and bands quantified using the Gel-Doc system (Bio-Rad).

## Competing interests

The author(s) declare that they have no competing interests.

## Authors' contributions

DD carried out and contributed to the design of the mRNA stability and expression studies. SN-E carried out and analysed the CLSM work and some of the expression studies. CHT and KO'B contributed the remainder of the expression work. JH assisted in the mRNA stability assays. DJ participated in the design of the CLSM studies and the data analysis for these and critically reviewed the manuscript. DP helped conceive and assisted in the design of the study, contributed methods and reviewed the manuscript. CMS helped conceive and assisted in the design of the study, contributed techniques for the mRNA stability work and critically reviewed the manuscript. SS helped conceived of the study, coordinated its design and implementation and drafted the manuscript.
